# Dry eye disease and psychosomatics—benefits of mind-body therapy for dry eye disease

**DOI:** 10.3389/fmed.2025.1600258

**Published:** 2025-08-20

**Authors:** Cong Zhao, Xiang Li

**Affiliations:** ^1^Eye School of Chengdu University of Traditional Chinese Medicine, Chengdu, China; ^2^Key Laboratory of Sichuan Province Ophthalmopathy Prevention & Cure and Visual Function Protection with TCM Laboratory, Chengdu, China; ^3^Retinal Image Technology and Chronic Vascular Disease Prevention & Control and Collaborative Innovation Center, Chengdu, China

**Keywords:** ocular surface, psychological stress, inflammation, biopsychosocial model, psychoneuroimmunology

## Abstract

Dry eye disease (DED) is a chronic inflammatory condition with increasing prevalence. Current treatment strategies, including artificial tears and anti-inflammatory agents, often fail to fully relieve ocular discomfort or meet patients’ broader clinical needs. Psychosomatic medicine is grounded in the biopsychosocial model of disease. Epidemiological studies suggest that DED is influenced by a range of physiological, psychological, and social factors. Psychoneuroimmunology (PNI) may play a key role in the interplay between DED and mental health. Therefore, DED may be a psychosomatic disease, and its management should follow a multifaceted approach that considers both molecular-level mechanisms and broader psychosocial factors. The efficacy of mind-body therapies (MBT) in psychosomatic disorders has been widely recognized in recent years, yet there is still a wide scope for exploration in DED. This review explores the psychosomatic aspects of DED, highlights its subjective symptom burden, and discusses the potential benefits and mechanisms of MBT as an adjunctive therapy, offering new perspectives for its comprehensive management.

## 1 Introduction

Dry eye disease (DED) is a chronic condition characterized by an imbalance in tear film homeostasis with a vicious cycle of ocular surface inflammation as the central mechanism, with a high prevalence worldwide (1–3). Common management options for DED include artificial tears, topical anti-inflammatory agents (such as corticosteroids, cyclosporine, and lifitegrast), ocular surface physiotherapy, and surgical interventions. However, a considerable proportion of patients fail to achieve satisfactory symptomatic relief (4). The significant physical and psychological burden caused by DED remains a serious public health issue (5, 6).

It is noteworthy that most current treatment approaches primarily target local ocular pathology, while giving insufficient consideration to patients’ psychosocial experiences and environmental influences. Recent evidence from basic science and epidemiological studies increasingly supports the classification of DED as a lifestyle-related condition. Various socio-environmental factors, including occupational stress, culture background, socio-economic status, exposure to conflict, and digital device use, have been implicated in the development and progression of DED (7–11). Mental health is also strongly associated with DED, and this effect is bidirectional (12). A meta-analysis of 2,980,026 individuals comparing the prevalence of depression and anxiety between patients with DED and healthy controls found that DED was significantly associated with a higher risk of depression (OR = 2.92, 95% CI: 2.13–4.01, *p* < 0.00001) and anxiety (OR = 2.80, 95% CI: 2.61–3.02, *p* < 0.00001) (13). The association between negative psychological states and subjective DED symptoms appears stronger than that between psychological factors and objective clinical signs (14). Additionally, the risk of DED is higher in depressed patients compared to the general population (15). These findings suggest that DED is an ocular condition with strong relevance to the field of psychosomatic medicine.

Psychosomatic ophthalmology emerged in the aftermath of the Second World War when patients presented with symptoms that could not be explained by physiological findings, and it was subsequently discovered that psychological factors may be involved in many eye diseases (16). In traditional biomedical models, the human body is explained as an extremely complex physicochemical machine. George Engel—an internist, psychiatrist, and psychoanalyst—proposed, based on clinical observations, that this mechanistic model was insufficient to explain the full complexity of human health and disease (17).

He emphasized that the environment and the individual together form a dynamic, holistic system that shapes both physiological and pathological processes. He proposed that the environment and the organism constitute a dynamic developmental holistic system that shapes both pathological and physiological processes. In clinical practice, many patients with DED experience significant subjective symptoms despite the absence of identifiable organic pathology (2). This ocular discomfort, which is not directly related to biological indicators, has led to a focus on the association between psychosomatics and ophthalmology and attempts at MBTs. Research has shown that some MBTs can alleviate ocular discomfort and improve DED symptoms, such as cognitive behavioral therapy (CBT)-based exercise programs and strength interventions (a form of positive psychology) (18, 19).

Although several studies, have explored psychosomatics factors in DED, the specific mechanisms linking MBT to DED remain insufficiently understood. This review aims to elucidate the psychosomatic underpinnings of DED, examine the challenges in evaluating subjective symptoms, and assess the therapeutic potential and underlying mechanisms of MBT in its treatment.

## 2 DED and psychosomatics

### 2.1 DED and biopsychosocial models

During the development of conventional medicine, psychosocial factors were often overlooked, which does not mean that they ignored the suffering of their patients, but they tended to downplay their scientific relevance (20). A similar trend has also been observed in traditional ophthalmic practice. Ophthalmologists tend to focus more on the objective and quantitative elements of the disease, such as clinical features and examination reports from relevant medical devices (e.g., slit lamp, ophthalmoscope, phoropter), and diagnose and treat it, usually through eye drops or surgery (21, 22).

Grinker coined the term biopsychosocial to emphasize the influence of socio-environmental factors on disease, a concept later expanded by Engel (23, 24). Engel proposed the biopsychosocial model of disease, arguing that the human body is more than a machine made of cells, tissues, and organs. It also exists within broader environments, including family, society, and ecosystems (25). Numerous studies have demonstrated that DED fits the biopsychosocial model better than the machine model. The following discussion explores this bidirectional relationship—how DED affects mental health and how psychological states, in turn, influence DED.

#### 2.1.1 Impact of DED on mental health

DED and mental health are closely linked and influence each other (26). DED causes a significant decline in physical and mental health-related quality of life and is also associated with an increased prevalence of depression and anxiety (27, 28). Notably, DED symptoms had a more significant impact on mental health than DED signs. Studies have found a stronger connection between DED symptoms and subjective well-being levels than signs (29, 30). In a study that evaluated the correlation between symptoms of DED and other factors, Galor et al. (31) found that dry eye symptoms were more strongly associated with non-ocular pain, depression, and post-traumatic stress disorder than with tear film parameters, one of the most significant clinical references used to diagnose DED. The negative impact of dry eye disease on mental health will be elaborated in detail in “Subjective Symptom Challenges in DED.”

#### 2.1.2 Impact of mental health on DED

**Figure 1:**
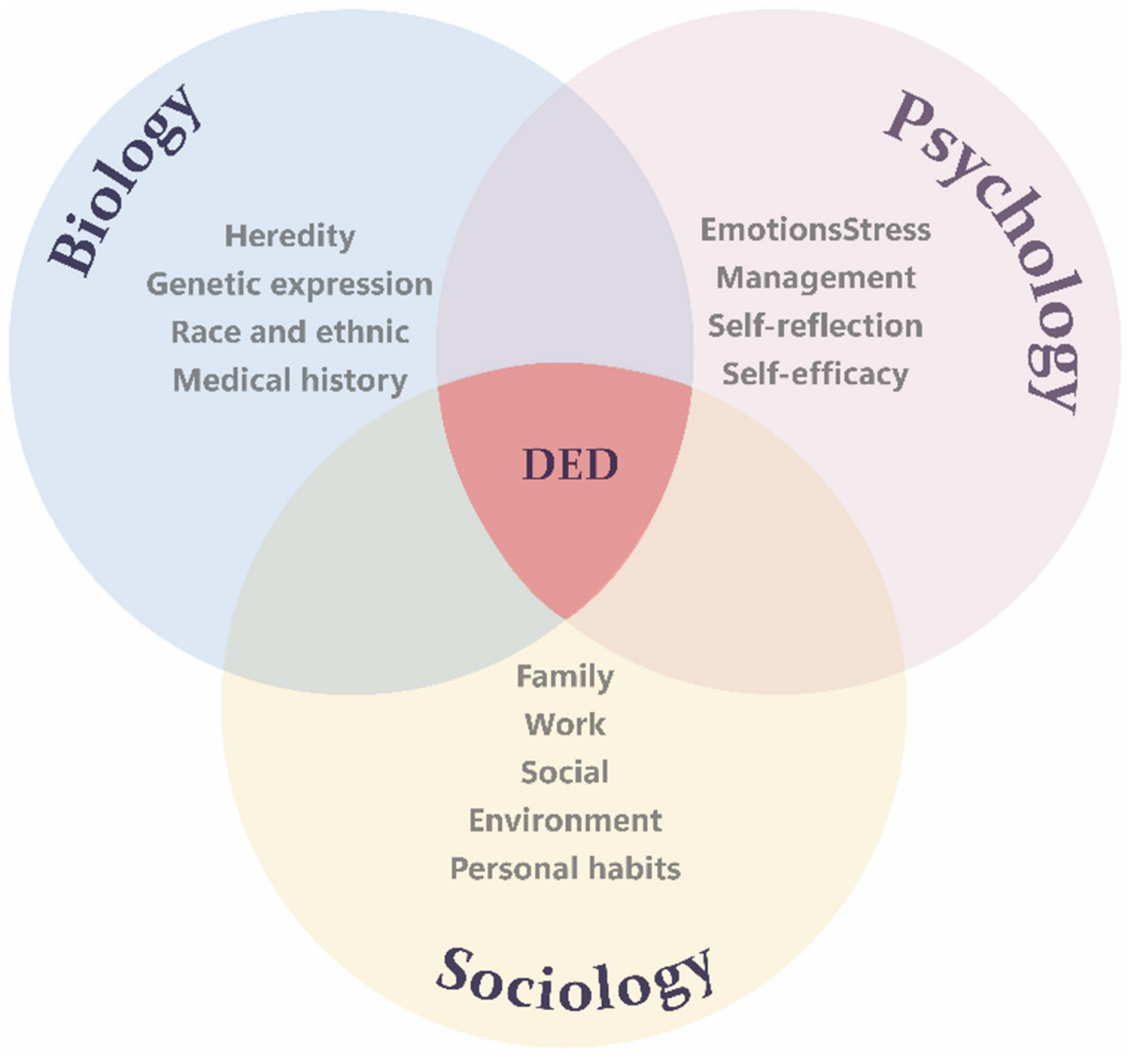
Main issues that contribute, according to the biopsychosocial model, to the pathogenesis of DED. The biopsychosocial model of DED encompasses three key dimensions: the biological dimension includes heredity, gene expression, race, ethnicity, and medical history; the psychological dimension covers emotional state, stress regulation, self-reflective thinking, and self-efficacy; the social dimension comprises family structure, work environment, social context, and personal behavior patterns. The overlapping areas of these dimensions illustrate how they interact to drive the onset and progression of DED.

Psychological distress is increasingly recognized as a significant risk factor in the development of DED. For example, one study comparing Schirmer test and tear break-up time scores of depressed individuals and healthy controls found that depressed patients showed reduced tear production function and tear film stability—both core features of DED (32). In addition, social and environmental factors can also impact the onset of dry eye disease by influencing mental health. The Tear Film & Ocular Surface Society (TFOS) Workshop presented a report on the impact of social challenges on the ocular surface (33). The report indicates that individual variability, social environments, and lifestyle choices are directly or indirectly related to ocular surface health. Ocular surface health is directly related to the development of DED (34). In biopsychosocial models, the crosstalk of different factors may alter the risk of developing DED. For example, advanced age is a significant risk factor for DED (12, 35, 36), but some evidence in recent years has negated the linear relationship between age and DED. Several studies have reported a high incidence of DED in children and young adults, indicating the relevance of other non-age-related risk factors, including gender, genetics, and exogenous variables (37–40). These factors contribute to the trend towards a lower age prevalence of DED. Furthermore, the same external factors can produce different mental projections and trigger different outcomes depending on individual experiences. Studies have reported that social media may affect adolescents’ perceived self-image, increase social anxiety, and affect self-confidence (41). This negativity may partially explain why adolescents with frequent screen exposure are more likely to develop depressive symptoms (42). Conversely, older adults’ exposure to social media may be beneficial, including reduced loneliness and creating social connections in both assisted and independent living communities (43, 44). This negative or positive mood can have different effects on DED. Figure 1 attempts to show the most crucial factors involved in the pathogenesis of DED according to the modern psychosomatic view.

### 2.2 DED and psychoneuroimmunology

The mechanisms of interaction between DED and biopsychosocial factors are unclear, which may involve PNI. The chronic pain and neurological disorders caused by DED may contribute to depression and anxiety (45). Additionally, the mechanism by which psychological stress affects the eye may involve a complex interplay between inflammatory responses and neurological disorders (46).

#### 2.2.1 Anatomical connections between DED and cognitive and emotional areas of the brain

Receptors in the eye and periocular tissues convert potential or actual damage to the tissues into electrical signals, transmitting this information to the trigeminal ganglion (TG) (47, 48). The majority of ocular surface-responsive TG neurons terminate in two spatially discrete regions of the inferior trigeminal brainstem nucleus complex: the transition region between the caudal interpolates (Vi) and caudal (Vc) (Vi-Vc transition), and the Vc/upper cervical cord junction (VcC1 region) (49–51). Ocular neurons located in the Vi-Vc transition and VcC1 regions project to the parabrachial area and posterior thalamus, rather than the main sensory thalamic areas (52). These areas have strong connections with the amygdala, insular cortex, and other limbic brain regions, which are particularly important for encoding the emotional experience and cognition of nociception (53).

Studies have shown that stimulation of the insula cortex triggers nociception in ocular tissues such as the cornea (54). In contrast, no sensation is produced in the eye after electrical stimulation of the brain’s primary somatosensory cortex, even though the brain’s primary somatosensory cortex can receive signals from the eye (e.g., bright light stimulation) (55). These studies suggest that among the relevant regions of the thalamus and cerebral cortex that produce nociception, the eye has a stronger connection to the emotional and autonomic aspects of the region than to the sensory-discriminative aspects of the region (56, 57). This means that DED-induced eye discomfort may trigger negative emotions such as depression and anxiety by stimulating these brain regions and that this effect works bi-directionally.

#### 2.2.2 Inflammatory responses in DED and psychological disorders

DED is an inflammatory disorder, and the development of many psychological disorders, including depression and anxiety, is associated with a neuroimmune inflammatory response, all of which involve multiple inflammatory factors (58, 59).

Recent evidence suggests that immune system dysregulation is associated with the pathogenesis of major depressive disorder (MDD) (60). A meta-analysis involving 22 studies (20,791 participants) found significant associations between concurrent depression and C-reactive protein (CRP) [*n* = 7; correlation coefficient *r* = 0.12; 95% confidence interval (CI) = 0.04 to 0.19] as well as interleukin-6 (IL-6) (*n* = 7; *r* = 0.17; 95% CI = 0.10 to 0.24). Additionally, a systematic review including 69 studies revealed that patients with MDD had higher levels of IL-6 and tumor necrosis factor-α (TNF-α) in cerebrospinal fluid and brain parenchyma compared with the control group [standardized mean difference (SMD) was 0.37, 95% CI: 0.17–0.57 and SMD 0.58, 95% CI 0.26–0.90, respectively] (61, 62). Elevated levels of pro-inflammatory cytokines may be associated with blood–brain barrier (BBB) damage and altered cognitive function. Matrix metalloproteinases-9 (MMP-9) have been found to play a critical role in BBB damage in various brain-related diseases, such as acute ischaemic stroke and traumatic brain injury (63, 64). The possible mechanism is that MMP-9 is involved in the degradation of tight junction proteins (TJPs) and the basement membrane of the BBB (65). The integrity of the BBB is essential for maintaining brain microenvironmental homeostasis, and damage to the BBB leads to the entry of large amounts of inflammatory cytokines and other neurotoxic substances into the brain, inducing neuroinflammation and damaging brain neurons (66). Damage to neurons in the prefrontal cortex, hippocampus, and amygdala, the areas of the brain associated with emotional and behavioral control, and the resulting reduced availability of neurotransmitters can lead to depression (67). In addition, neuroinflammation is also associated with chronic stress-induced social and cognitive changes. A study found that activation of the MMP-9 signaling module in the CA1 region of the hippocampus in mice was involved in the development of depressive-like behaviors (68). MMP-9 inhibitors prevented stress-induced deficits in social exploration, social memory, and CA1-dependent cognitive deficits (69).

The inflammatory response is a central factor in the pathogenesis of DED. TFOS Dry Eye Workshop II (DEWS-II) proposes that every type of dry eye, regardless of its triggers, eventually enters a vicious cycle pathway formed by tear hyperosmolarity and a series of inflammatory events that continue the DED state (59). Critical to this process is the expression of IL-1β, IL-6, and TNF-α, which are involved in triggering a cascade of events, including transcriptional activation of genes encoding inflammatory MMPs (especially MMP-9) and pro-apoptotic factors (59, 70–72). Expression of MMP, a protein hydrolase involved in wound healing and inflammation, plays a central role in ocular surface epithelial barrier damage (73). MMP is associated with reduced expression of glycocalyx mucins, apoptosis of surface epithelial cells, and loss of cup cells (74, 75). These processes disrupt intercellular epithelial junctions, disrupting the epithelial barrier and elevating tear osmolarity (76). Studies have shown a positive correlation between MMPs (including 1, 3, 9, and 13) and elevated tear osmolality, and among these proteases, MMP-9 is of central importance in the hyperosmotic stress response (77, 78).

Both psychological disorders and DED are closely associated with inflammatory responses. The mechanisms may be related to the regulation of the autonomic nervous system (ANS).

#### 2.2.3 Autonomic nervous system regulation

The ANS is one of the major neural pathways activated by stress (79). Stress activates the sympathetic nervous system (SNS) and inhibits the parasympathetic nervous system (PNS) (80). The persistence of stress causes the SNS to be in a state of prolonged activation and it lacks the normal counterbalance of the PNS. As a result, peripheral levels of catecholamines increase, and levels of acetylcholine decrease (81). Acetylcholine is anti-inflammatory, which in turn increases the levels of pro-inflammatory cytokines such as tumor necrosis factor (TNF), interleukin-1 (IL-1), IL-6, and interferon, leading to an inflammatory state (82). These inflammatory responses are strongly associated with depression and DED.

**Figure 2:**
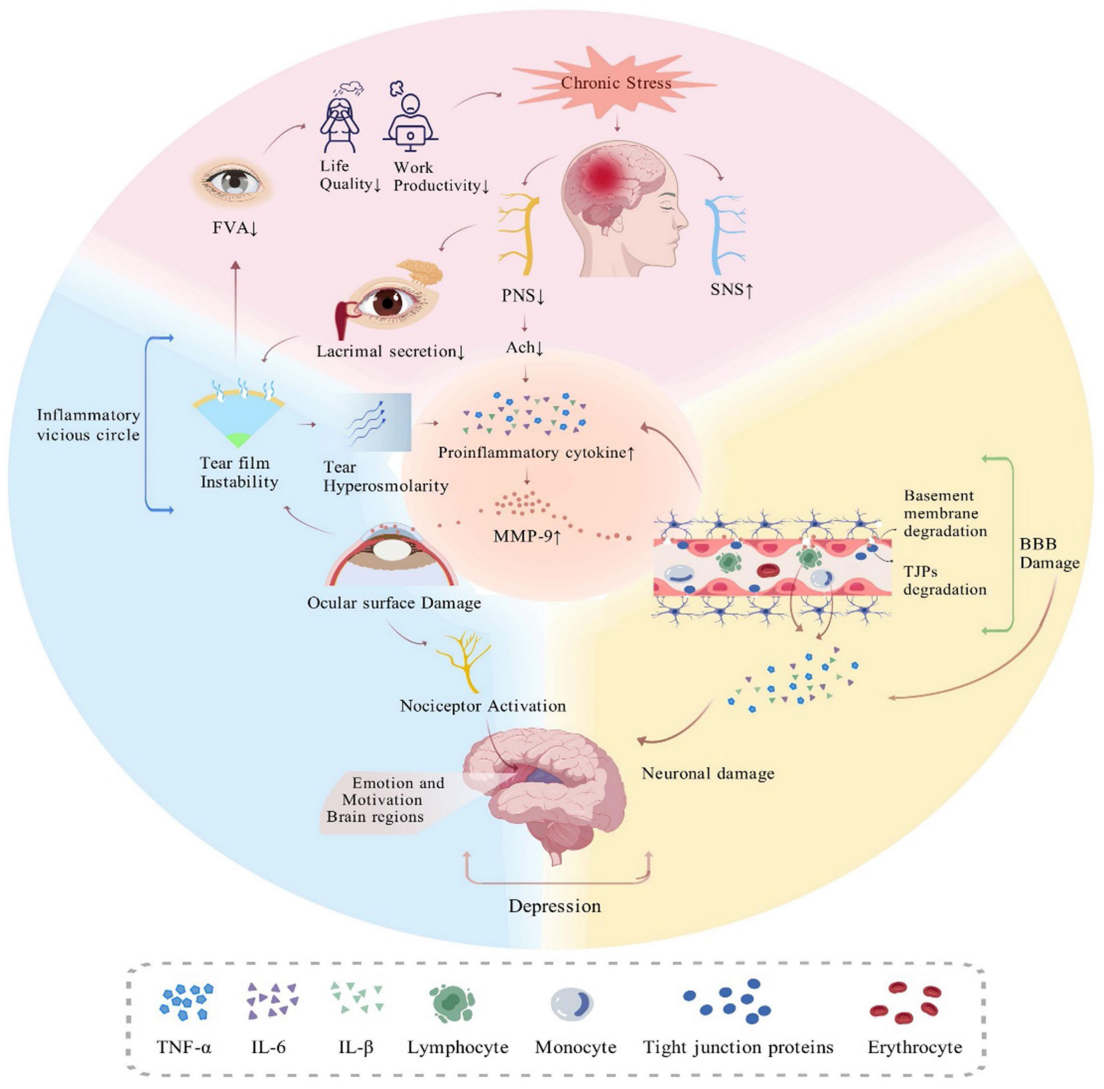
DED and PNI (created with biogdp.com). The close anatomical connection between DED and the cognitive and emotional regions of the brain means that the subjective symptomatic discomfort triggered by DED can cause more severe negative emotions and psychological stress. The long-term negative impact of DED on quality of life and work can lead to persistent stress. The disruption of the ANS by chronic stress disrupts the balance between the SNS and PNS, inducing an inflammatory response. The disruption of the BBB by inflammatory factors further aggravates the inflammatory response and induces neuronal damage in the brain, and neuronal damage to cognitive and affective regions is closely associated with the development of MDD. In DED, on the one hand, reduced parasympathetic activity leads to suppression of tear gland secretion and disruption of tear film homeostasis, while the inflammatory response promotes an imbalance of tear film homeostasis; on the other hand, an imbalance of tear film homeostasis induces a vicious cycle of inflammation on the ocular surface, exacerbating ocular discomfort, and this nociception is transmitted to the sensory regions of the brain, further decreasing the quality of life and work, and triggering negative emotions and psychological stress.

In addition, recent studies have found a significant correlation between DED symptoms and autonomic nerve activity and a closer association between parasympathetic nerve activity and DED symptoms (83, 84). The ANS may be involved in the pathogenesis of DED by regulating tear film homeostasis (85). The tear film is essential for protecting the ocular surface and maintaining normal visual function, and an imbalance in tear film homeostasis is a key characteristic of DED (86). Studies have shown that muscarinic receptor antagonists, which inhibit parasympathetic activity, reduce secretion from the lacrimal glands (87). In contrast, muscarinic receptor agonists are effective at enhancing tear production and improving tear film stability (88). Significantly, the lacrimal gland receives innervation from both sympathetic and parasympathetic nerves, but the effect of sympathetic nerves is less effective than that of parasympathetic nerves in promoting tearing (89) (Figure 2).

### 2.3 Basic mind-body care: autonomic nervous system regulation and healthcare provider satisfaction enhancement

Jutta suggested training in “basic mind-body care” for ophthalmologists. Mind-body care not only facilitates physician-patient communication but also alleviates stress and contributes to disease recovery by regulating the ANS. Lin et al. (90) evaluated the impact of aromatherapy on the recovery of the autonomic nervous system to baseline levels in adolescents after exercise through the calculation of heart rate variability (HRV). The results showed that, compared with the control group, adolescents who received aromatherapy had better regulation of autonomic nervous function, and the therapy exerted a significant stress-reducing effect. Similarly, Inbaraj et al. (91) explored the protective effect of mind-body therapy (yoga) on autonomic nervous function in breast cancer patients undergoing chemotherapy by means of HRV. They found that yoga could serve as a potential auxiliary intervention to improve cardiac health and prevent cardiovascular-related diseases in breast cancer patients receiving anthracycline chemotherapy. Although there is currently a lack of relevant research on improving the autonomic nervous system function in DED patients through mind-body care, given that improving autonomic nervous system function can effectively reduce the body’s inflammatory response, it undoubtedly has positive significance for the recovery of DED patients. The main contents and objectives of this training are as follows: to view the individual and the disease holistically through the biopsychosocial model, to promote effective communication, and to enhance job satisfaction among ophthalmologists. Furthermore, doctors are expected to take care of themselves and use their resources and strengths to maintain a long-term enjoyment of life and work and to avoid burnout. Wiek and Emmerich (92) also proposed that psychosomatic medicine can be a tool for building a working alliance between doctors and patients and that ophthalmologists should guide their patients in coping with their disease through a resource-oriented approach. Studies have shown that resource-oriented psychosocial interventions can achieve therapeutic outcomes and/or symptom reduction by empowering patients to participate more actively in their treatment and learn new skills to deal with their condition (93). In particular, as a lifestyle-related disease, the subjective initiative of DED patients, such as avoiding risk factors and bad habits and seeking psychological care, is very important in the clinical management process.

Psychosomatic medicine not only facilitates patient recovery but also enhances the professional satisfaction of healthcare providers, with the underlying rationale as follows: at the conceptual level, psychosomatic medicine emphasizes the “biopsychosocial” holistic perspective, which not only focuses on the physiological mechanisms of diseases but also attaches greater significance to the impact of psychological states and social environments on individual health (including that of healthcare providers themselves). This perspective enables healthcare providers to transcend the simplistic “disease-centered treatment” framework, thereby recognizing that work-related issues such as doctor-patient conflicts and occupational stress are not isolated phenomena. For example, patients’ anxiety may exacerbate the complexity of diagnosis and treatment, while healthcare providers’ emotional exhaustion can compromise the quality of medical services (94, 95). Such a holistic understanding allows healthcare providers to respond to work challenges more rationally, mitigating the frustration arising from “inability to resolve all problems” and further strengthening their recognition of professional value. At the practical level, psychosomatic medicine-related training (e.g., “basic mind-body care”) equips healthcare providers with specific self-regulation and interaction skills. For instance, ANS regulation techniques covered in the training (such as mindful breathing and progressive muscle relaxation) can alleviate acute work-related stress (e.g., prolonged surgeries, unexpected medical incidents) and reduce the incidence of occupational burnout (96, 97); meanwhile, resource-oriented communication skills (e.g., empathetic listening, guiding patients to articulate psychological needs) can enhance the quality of doctor-patient interactions, minimize conflicts stemming from communication barriers, and reduce the accumulation of negative emotions in the workplace (98). When healthcare providers can effectively manage their own states and establish more harmonious relationships with patients, “emotional depletion” in work decreases while “professional fulfillment” increases, which inherently elevates their job satisfaction.

In summary, a comprehensive understanding of the relationship between MBT and DED can help in communicating with patients, establishing a good doctor-patient relationship, and effectively executing treatment plans. It also helps to improve doctors’ job satisfaction.

### 2.4 Exploring the relationship between conventional dry eye diagnostic indicators and psychosomatic symptoms

DED is often diagnosed using objective tests such as tear film breakup time TBUT, Schirmer’s test, and corneal staining scores. These tests help evaluate tear stability, tear production, and damage to the corneal surface. However, in clinical settings, these objective indicators do not always match the patient’s reported symptoms, such as foreign body sensation, burning, or eye pain (2). The prevalence of DED can vary greatly depending on whether the diagnosis is based on symptoms alone or on a combination of symptoms and signs (12). This mismatch becomes more complex when psychosomatic symptoms, such as anxiety, depression, chronic pain, or sleep problems, are involved. Ashena et al. (99) proposed that individuals with depression and anxiety may interpret ocular sensations differently from healthy controls. Their emotional state can affect how they report symptoms. In some cases, psychological stress may increase sensitivity to pain through central nervous system pathways. At the same time, some patients with severe corneal staining report only mild discomfort (2). This inconsistency between signs and symptoms supports the idea that psychological and neurological factors play a role in DED. Moreover, imbalances in the autonomic nervous system may link mental health and tear secretion. Chronic stress can reduce parasympathetic activity and increase sympathetic arousal, which may affect tear gland function and eye inflammation (81, 87).

In conclusion, conventional tests are still important for diagnosing DED, but their results may be influenced by psychological conditions. A more complete evaluation should include psychosocial factors. This is especially important for patient-centered care and developing more precise treatment strategies.

## 3 Subjective symptom challenges in DED

In ophthalmic practice, it is estimated that one-third of patients present with ocular symptoms for which no organic cause can be found. Unclear biological targets complicate disease management (100). Previously, increasing tear stability was considered an effective method of alleviating dry eye clinical symptoms. However, it has been shown that some dry eye sufferers are resistant to having symptoms subjectively improved (101, 102). Here we review the manifestations of subjective symptoms that cause discomfort in patients with dry eye disease.

### 3.1 Ocular surface discomfort and decreased functional visual acuity

Utility assessments have estimated that serious DED symptoms are as debilitating as severe angina (103). Of the ocular symptoms associated with DED, ocular nociception is the most common, and its presentation is complex and varied. The short-form McGill Pain Questionnaire, which uses 15 descriptors (throbbing, shooting, stabbing, sharp, cramping, gnawing, burning, aching, heavy, crushing, tearing, fatigue, nausea, fearful, and/or punishing-cruel) to describe the severity (mild, moderate, severe) and type of pain, was used in one study to examine how DED symptoms are presented (104). The study found that 82% of patients mentioned at least one or more of these traits when describing their ocular symptoms (105). The TFOS DEWS II pain and sensation report proposed that the majority of patients do not express pain directly in clinical practice but describe their sensations in terms of itchiness, discomfort, and dryness and that this unpleasant dry eye sensation should be regarded as a specific form of eye pain that occurs in this particular disease (106). Ocular pain can be classified as nociceptive or neuropathic pain based on etiology, duration, or clinical features (107). When tissues are actually or potentially injured, pain receptors rapidly transmit signals that activate the inflammatory response and the immune system to protect the tissues (108). However, exceeding the regular tissue healing time can make the pain chronic or persistent and difficult to treat (109). Neuropathic pain, also described as pathological or biologically worthless pain, manifests as nociceptive sensitivity, including injurious responses to non-toxic stimuli and abnormal hypersensitivity responses to stimuli (110).

The management of chronic and neuropathic ocular pain in patients with DED is a tough challenge for ophthalmologists (111). Commonly used clinical analgesic regimens such as opioids and non-steroidal anti-inflammatory drugs are rarely used in patients with DED due to their high risks and limitations such as drug addiction, tolerance, and side effects like induction of renal damage (112–114). As a consequence, many eye drops with anti-inflammatory, inflammatory modulating, and neurotrophic effects have been developed. However, these drops have limited pain relief and have certain limitations, such as long-term use of anti-inflammatory drugs can induce glaucoma and cataracts (115); immunomodulators are expensive and have a long latency period, which reduces patient compliance (116, 117); and autologous serum tears are limited in clinical use due to high standards of preparation, transport, and storage (118). Thus, other safe, effective, and cost-effective therapies are necessary.

In addition, ocular itching is a specific DED pain manifestation. Itch has many of the same elements as pain, such as the fact that neurons in both are sensed by small-diameter, unmyelinated C fiber in the dorsal root ganglion and trigeminal ganglion (TG) (119). Unlike the pain sensation, the sensation of itching and the associated scratching impulse leads to an itch-scratch cycle. Long-term, chronic itching can lead to habitual, almost compulsive scratching, exacerbating local tissue damage and inflammatory responses (120). The deleterious effects of chronic itch on quality of life are comparable to those of chronic pain, and it can cause debilitating effects such as irritation and sleep disturbances that can lead to clinical depression (121).

The ocular discomfort and tear-related aberration changes caused by DED can interfere with normal visual function (12, 122). In clinical practice, many patients with DED often complain of difficulty seeing despite good vision. Kaido et al. (123) compared the vision-related parameters of DED patients and normal subjects using a functional visual acuity (FVA) measurement algorithm, and the results showed that there was no difference in initial vision, average response time, and blink frequency, but FVA and visual maintenance ratio were reduced in the dry eye patients compared to the normal group. These DED patients who temporarily do not show significant organic visual impairment are still affected by ocular discomfort and functional vision loss, which reduces the quality of life and work, especially the heavy financial loss due to reduced work productivity, which causes both physiological and psychological stress and impairs somatic and mental functioning (124).

### 3.2 Sleep disorders

The negative effects of DED and sleep disorders are bidirectional. A meta-analysis involving 21 studies (419,218 participants) found that patients with dry eye disease had a significantly higher risk of sleep disorders than those without dry eye disease [relative risk (RR) = 2.20, 95% confidence interval (CI): 1.78–2.72, *p* < 0.001]. They also had a higher risk of sleep insufficiency compared with the control group (RR = 3.76, 95% CI: 3.15–4.48, *p* < 0.001), and the prevalence of excessive daytime sleepiness was higher than that in the control group (RR = 5.53, 95% CI: 3.83–7.18, *p* < 0.001) (125). Dry eye patients may experience depression, pain, and nighttime eye exposure, all of which disrupting their sleep (126, 127). In addition, sleep disorders can exacerbate dry eye symptoms (128, 129). Research has shown that chronic sleep disorders can lead to autonomic nervous system dysfunction, which affects the parasympathetic function of the lacrimal gland and reduces tear production (89, 130).

## 4 DED and mind-body therapies

MBT explores the relationships between the brain, mind, body, and behavior to improve physical function and promote health through integrated mental and physical training (131). The effectiveness of MBT has been demonstrated in various medical fields, including holistic frailty prevention, stress management, chronic inflammation, pain relief, and mood disorders (132–136). And MBT offers distinct advantages regarding safety and cost-effectiveness (137, 138). Joint multidisciplinary research between public health, psychology, and ophthalmologists suggests that a positive mental state has positive implications for ocular surface health (139). Studies have proven that MBT such as CBT, exercise, massage, and mindfulness can help reduce dysfunctional and maladaptive thinking and behaviors and improve coping mechanisms, resulting in significant improvements in negative psychology (140).

The growing interest in MBT for DED has been notable. Li et al. (141) conducted a study using laughter exercises to intervene with patients experiencing symptomatic dry eye. They found that laughter exercises were comparable to 0.1% sodium hyaluronate in relieving subjective symptoms and were more effective in extending the time until non-invasive tear film breakup in dry eye patients with minimal corneal staining. Laughter therapy is a readily available, acceptable, and low-cost form of CBT that has been demonstrated to enhance mental health, increase resilience, and improve overall quality of life (142). In addition, MBT not only improves DED symptoms but also enhances patients’ well-being and quality of life. Sano et al. (19) developed a 10-week CBT-based exercise program for office workers with dry eye symptoms and found that the means of dry eye-related quality of life scores and subjective well-being scale scores tended to increase after the intervention.

The benefits and potential mechanisms of mind-body therapies for DED are described below.

### 4.1 Neuromodulatory mechanisms of mind-body therapies associated with pain

DED-induced ocular pain is a complex issue. Research has shown that emotional states significantly influence pain perception; positive emotions can reduce pain, while negative emotions can exacerbate it (143). Interestingly, in certain situations, pain may be perceived as pleasurable, such as during a deep tissue massage, which can create a positive motivational experience (144). This phenomenon is closely tied to how we perceive pain, as it is often the emotional aspect of pain that determines its intensity and unpleasantness, making it feel painful (145, 146).

Studies have found that the damage observed in patients with chronic pain extends beyond the pain itself, which may be related to anatomical alterations within areas of the brain associated with pain-related cognition and emotion regulation. Several studies have observed grey matter loss and white matter bundle disruption-like changes in the frontal brain in the brains of chronic pain patients (147, 148). Excitingly, this alteration can be reversed with active intervention (149). Multiple studies have shown that MBT can enhance positive emotions by modulating brain activity while preventing depression, anxiety, and negative affect and reducing perceived stress, as well as promoting self-compassion (150). For example, CBT can increase neuronal activity in the right inferior frontal gyrus and the right dorsolateral prefrontal cortex to reduce pain (151); meditation and mindfulness can modulate the connectivity of circuits associated with emotional and cognitive function, such as increasing the volume of grey matter in the left anterior insula and improving the structural plasticity of white matter around the anterior cingulate cortex and the posterior cingulate cortex (152, 153); the prefrontal cortex is a flexible hub that regulates an individual’s negative emotions (154). Studies have found that tai chi increases the thickness of the frontal sulcus cortex in practitioners, while yoga increases the thickness of the left prefrontal cortex in practitioners (155, 156).

Traditional Western medical therapies have favored medication for pain over mind control. At present, the potential of MBT has been recognized. More and more studies are beginning to employ these techniques for stress reduction and pain control (154, 157). Multiple systematic evaluations and meta-analyses have reported that MBT is effective in improving nociception and physical functioning in a wide range of refractory pain-related disorders (157–160).

It is important to note that dry eye-related ocular pain may be either nociceptive or neuropathic. Mind-body therapies (MBT) may be more effective for neuropathic pain. However, if the pain is caused by medical procedures or diseases—such as LASIK-induced neuropathic corneal pain, nerve injury after chemotherapy or radiotherapy, viral infections, or diabetic neuropathy—MBT alone may have limited effect (161–164). Therefore, a clear diagnosis and classification of ocular pain should be made first. For patients with neuropathic pain, neurotrophic support and anti-neuropathic medications can be used. MBT can then be considered based on the individual’s condition. In complex cases, consultation with specialists in neuropathic pain may be needed to ensure a scientific and personalized treatment plan, and to improve the accuracy and effectiveness of intervention.

### 4.2 Regulation of inflammation and autonomic nervous system

Breaking the vicious cycle of inflammation in the ocular surface is crucial for treating DED. Studies have shown that MBT effectively reduces the activity of the NF-κB signaling pathway and decreases the expression levels of key inflammatory factors, such as IL-1, TNF-α, and IL-6 (165–169). These pro-inflammatory cytokines play a critical role in sustaining the inflammatory cycle in DED (170–172). They recruit numerous inflammatory cells (both innate and adaptive) to the ocular surface by releasing signals that trigger various responses. These signals may be soluble or membrane-bound and include chemokines and adhesion molecules (59). Chemokines bind to macrophages, dendritic cells, neutrophils, and activated T cells (173). Endothelial adhesion molecules receive and transmit signals that trigger downstream responses through binding to the cytoskeleton and various junction proteins (174). These inflammatory responses ultimately induce corneal and conjunctival apoptosis, leading to damage to ocular surface tissues, which further triggers imbalances in tear film homeostasis and tear hyperosmolarity, promoting a vicious cycle of inflammatory responses (59).

There is evidence that MBT has a modulatory function on ANS, such as meditation reduces resting systolic and diastolic blood pressure, increases resting heart rate variability, and increases resting cerebral blood flow (175–178). Notably, MBT can enhance autonomic function in response to stress. Research has found that mindfulness improves stress-induced emotional and physiological challenges and leads to faster physiological and emotional recovery after acute stressful tasks (179, 180). Stress is strongly associated with mental health and ocular surface health. In addition, MBT can reduce threat arousal by shifting the autonomic nervous system to parasympathetic dominance through slowing and/or modulating breathing (181). Parasympathetic activation promotes tear secretion and has positive implications for tear film stability (182). Research indicates that instability in the tear film can lead to alterations in optical quality and introduce higher-order aberrations, negatively impacting normal visual function. Enhancing tear film stability may mitigate these effects and positively influence the improvement of visual quality (12).

### 4.3 Regulation of sleep and emotional disorders

Improvements in emotional disturbances with MBT are associated with positive effects on affect, cognition, and brain network connectivity. A randomized controlled trial found that CBT reduced salience network connectivity in the lingual gyrus of the brain in depressed patients in a rumination state, modulated neurocognitive function during depressive rumination, and enhanced the ability to sustain attention to the body (183). Another cross-sectional study found that in older women with at least 8 years of yoga practice experience, functional magnetic resonance imaging showed that their brains exhibited greater intra-network anteroposterior brain functional connectivity of the Default Mode Network. This alteration helps to maintain brain connectivity and self-awareness to maintain a healthy state (184).

Similarly, MBT had a significant positive effect on sleep quality (185, 186). A meta-analysis encompassing 61 randomized controlled trials revealed that, compared with the control group, mindfulness exercise training significantly improved sleep quality [standardized mean difference (SMD) = −0.794; 95% confidence interval (CI): −0.994 to −0.794, *p* < 0.001, *I*^2^ = 90.7%]. Moreover, practicing at least twice a week (SMD = −0.793; 95% CI: −0.868 to −0.718; *p* < 0.001) was more effective than practicing once a week (SMD = −0.687; 95% CI: −0.804 to −0.570; *p* < 0.001) (187). The mechanisms involved may be related to MBT’s interventions on mood, cognition, and brain function (188).

### 4.4 Limitations of mind-body therapy

Although MBT have shown some therapeutic potential in relieving dry eye-related pain, emotional distress, and sleep problems, their effects remain limited, especially in moderate to severe cases. In patients with strong inflammatory responses and meibomian gland dysfunction, dry eye is mainly driven by a vicious cycle of tear film instability and ocular surface inflammation. Since MBT focus on regulating the psycho-neuro-immune system, they may not be effective for these organic conditions. In addition, for some patients with neuropathic pain caused by nerve injury—such as after LASIK surgery, chemotherapy, or viral infection—MBT alone may not be sufficient to relieve symptoms. Therefore, in clinical practice, treatment plans should be personalized based on disease type, underlying mechanisms, and individual differences. Combining MBT with medications or other local treatments when appropriate may help achieve better outcomes.

## 5 Conclusion

This study explores the relationship between psychosomatic medicine and DED, emphasizing the significance and complexity of the biopsychosocial model of DED and that this complexity may be related to psychoneuroimmunological mechanisms. Psychosomatic medicine can serve as a tool for doctors and patients to co-manage DED, enhancing patients’ subjective initiative and increasing job satisfaction among healthcare workers.

This review summarizes the subjective symptomatic challenges of DED and the benefits and potential mechanisms of MBT as a treatment for DED, including improvements in pain, inflammation, emotional disturbances, sleep disorders, and regulation of ANS: anti-inflammatory and promotion of tear secretion (to reduce the negative impact of higher-order aberrations on visual function due to tear film instability). MBT can improve emotional and cognitive function, maintain attention, and mitigate distress by improving brain function and strengthening brain network connections. The application of MBT in DED is promising. However, there is currently limited research. Future systematic and large-scale studies are still needed to explore the effectiveness and pathophysiological mechanisms of MBT in DED.

In conclusion, DED is not only an oculopathy but also a comprehensive disease of the body and mind. Therefore, the management of DED requires a multifaceted consideration from the perspectives of the physiological, psychological, and social environment. And it is necessary to build a comprehensive treatment strategy that combines local organs with the whole body and mind.
